# The Need for Organisation

**Published:** 1918-12-07

**Authors:** 


### THE NEED FOR ORGANISATION.

A Bill to establish a Ministry of Health has been introduced in Parliament. In the nature of things it is wide in scope and void of detail. The thing is it has been introduced. The first step is taken. The new Minister is to take action conducive to the health of the people, including measures for the prevention and cure of disease, the treatment of physical and mental defects.

Prevention, Cure, Treatment. These are portentous words, and surely must portend a State Medical Service, for how else is the Minister to perform duties that include in their scope all of sanitary science and of medical and surgical treatment? "The training of persons engaged in Health Services"—here is weighty work and valuable, if well done. Surely no Minister ever had so great a call, such possibilities of power, as will be given to him who shall first assume the office. It is only temporarily, and whilst means are devised to disentangle the other than medical from the medical duties of the Local Government Board that all its powers and duties are transferred to the new Ministry. It is obviously out of the question for the duties in relation to fire brigades, piers and harbours, steam whistles and emigration to remain with the Minister of Health. These, with other matters, are noted in the first schedule for transfer to, presumably, either a new authority or a reorganised Local Government Board.

The medical profession is in revolution. It will be supremely interesting to see how the wheel will turn. If the medical profession wishes to guide the whirlwind and control the storm, wishes to be master of its own fate, it must organise itself now, take steps, make plans, for soon it will be too late. The medical profession has no collective voice, no organisation. The British Medical Association has its uses, but it does not speak for the profession as a whole; its heads are not regarded as representative, even by its own members. The General Medical Council has no authority to speak for the mass of medical men. It is apart from knowledge of the needs of the men in general practice, and has not concerned itself with general medical policy. There are other less important medical bodies, but none to lead, none to advise, none to negotiate with power and authority. If the medical profession is not united and ready to stand shoulder to shoulder it will be kept subordinate in future schemes, will be put under orders, will be exploited and stampeded, as it has been before. On what happens in the next few years will depend what the medical profession is to be in the future. It may be a subordinate and little-regarded body of men; it may be a powerful organisation of scientific men acting in concert and speaking with weight. The public is more concerned in the happenings and their result than the public knows, for it is certain that, if the profession is to be subordinate and little regarded, men of intellect and ability, worthy of regard, will stay out of it. If the profession is to be a powerful and highly-thought-of scientific organisation offering good and great rewards for honest and remarkable services, men of ability, men of great intellectual gifts and high ambition, will come in. In the first case the national health will be in the hands of such men as are content to be subservient and little considered. In the second case, in the hands of the best brains in the land.

The time is short, but there is time. The new day is dawning, but its work is not begun. It behoves the profession to arrange itself, to meet and discuss, to organise and make plans. Nothing can be done in these days without collective action and plans prepared in advance. Union is strength. Disorganisation soon becomes panic and rout and hopeless defeat. Medical men must show themselves willing to work for and with the community, but they have a right to a weighty voice as to how the work should be done and how it is to be paid. To have that voice they must be ready and able to bargain for due reward for their work in cash, kind, and consideration.

